# Can Machine Learning Correctly Predict Outcomes of Flexible Ureteroscopy with Laser Lithotripsy for Kidney Stone Disease? Results from a Large Endourology University Centre

**DOI:** 10.1016/j.euros.2024.05.004

**Published:** 2024-05-22

**Authors:** Carlotta Nedbal, Sairam Adithya, Nithesh Naik, Shilpa Gite, Patrick Juliebø-Jones, Bhaskar K. Somani

**Affiliations:** a University Hospital Southampton NHS Trust, Southampton, UK; b Urology Unit, Azienda Ospedaliero-Universitaria Delle Marche, Università Politecnica Delle Marche, Ancona, Italy; c Symbiosis Institute of Technology, Pune, India; d Manipal Academy of Higher Education, Manipal, India; e Department of Clinical Medicine, University of Bergen, Bergen, Norway; f Department of Urology, Haukeland University Hospital, Bergen, Norway

**Keywords:** Artificial intelligence, Ureteroscopy, Kidney calculi, Machine learning

## Abstract

**Background and objective:**

The integration of machine learning (ML) in health care has garnered significant attention because of its unprecedented opportunities to enhance patient care and outcomes. In this study, we trained ML algorithms for automated prediction of outcomes of ureteroscopic laser lithotripsy (URSL) on the basis of preoperative characteristics.

**Methods:**

Data were retrieved for patients treated with ureteroscopy for urolithiasis by a single experienced surgeon over a 7-yr period. Sixteen ML classification algorithms were trained to investigate correlation between preoperative characteristics and postoperative outcomes. The outcomes assessed were primary stone-free status (SFS, defined as the presence of only stone fragments <2 mm on endoscopic visualisation and at 3-mo imaging) and postoperative complications. An ensemble model was constructed from the best-performing algorithms for prediction of complications and for prediction of SFS. Simultaneous prediction of postoperative characteristics was then investigated using a multitask neural network, and explainable artificial intelligence (AI) was used to demonstrate the predictive power of the best models.

**Key findings and limitations:**

An ensemble ML model achieved accuracy of 93% and precision of 87% for prediction of SFS. Complications were mainly associated with a preoperative positive urine culture (1.44). Logistic regression revealed that SFS was impacted by the total stone burden (0.34), the presence of a preoperative stent (0.106), a positive preoperative urine culture (0.14), and stone location (0.09). Explainable AI results emphasised the key features and their contributions to the output.

**Conclusions and clinical implications:**

Technological advances are helping urologists to overcome the classic limits of ureteroscopy, namely stone size and the risk of complications. ML represents an excellent aid for correct prediction of outcomes after training on pre-existing data sets. Our ML model achieved accuracy of >90% for prediction of SFS and complications, and represents a basis for the development of an accessible predictive model for endourologists and patients in the URSL setting.

**Patient summary:**

We tested the ability of artificial intelligence to predict treatment outcomes for patients with kidney stones. We trained 16 different machine learning tools with data before surgery, such as patient age and the stone characteristics. Our final model was >90% accurate in predicting stone-free status after surgery and the occurrence of complications.

## Take Home Message

Machine learning (ML) can be used in a clinical settings to accurately predict outcomes after training on pre-existing data sets. Our ML model achieved accuracy of >90% in predicting stone-free status and complications for ureteroscopic laser lithotripsy. These findings provide a foundation for the development of an accessible predictive model for this setting.

## 1 Introduction

The prevalence of urolithiasis, which has been assessed as ∼1–20%, is increasing worldwide, mainly because of the association with metabolic disorders and population aging [1]. With a high risk of recurrence, which can affect more than a quarter of patients with urinary stones in the first 5 yr after diagnosis, the burden of urolithiasis is increasing worldwide. With growing interest in prevention and medical optimisation of patients affected by this condition, surgical treatment remains a primary importance [2].

The European Association of Urology guidelines recommend various options that are suitable for first-line treatment of different stone types [3]. Shockwave lithotripsy and flexible ureteroscopy with laser lithotripsy (fURSL) are considered the gold standard for stones smaller than 2 cm, while percutaneous nephrolithotomy (PCNL) is recommended for larger calculi. While URSL appears to be a feasible option for even large stones in the future, limitations currently persist [4]. Nevertheless, as technological innovations improve instrument quality, leading to shorter operative times and faster laser lithotripsy, there is growing evidence supporting the role of fURSL for higher stone burdens [5].

Various preoperative and intraoperative factors have been identified as potential risk factors for postoperative complications and failure to achieve stone-free status (SFS) in a single procedure [6]. A positive preoperative urine culture is a well-recognised risk factor for postoperative infectious complications [7], while the size, location, shape, and hardness of the calculus can limit retrograde surgery and correlate with postoperative events [8]. Despite this knowledge, uncertainties remain regarding the precise correlation between preoperative and postoperative features, hindering the development of efficient tools for predicting fURSL outcomes.

The past few years have seen substantial interest in incorporating machine learning (ML) into health care because of its unparalleled possibilities for improving patient care and results [9]. A particularly noteworthy ML application is as a tool for predicting postoperative outcomes [10]. The ability to anticipate postoperative complications, predict recovery paths, and assess overall patient wellbeing represents a transformative change in surgical health care [11]. Our study objective was to develop an ML model for automated prediction of postoperative outcomes after fURSL using data for preoperative patient characteristics.

## 2 Patients and methods

### 2.1 Data collection

We analysed data from a cohort of adult patient treated with fURSL for urolithiasis between March 2012 and December 2018. Operations were performed or supervised by a single experienced surgeon (B.K.S.) in a tertiary endourology centre. All patients with a positive urine culture had protocol-based treatment on the basis of antibiotic sensitivity. A repeat urine culture was performed for most but not all patients if a positive culture result was soon after initial antibiotic therapy. For most patients, antibiotic therapy involved a 7–10-d oral regimen.

The standard protocol for all cases involved a consent process for educational purposes routinely obtained before each procedure and collection of completely anonymised data. The kidney database encompasses 859 records, capturing preoperative details (age, sex, anatomic variations, comorbidities, stone size, total stone burden [cumulative length], stone number and locations, urine culture results, presence of a preoperative urinary drain), intraoperative information (procedure duration, anaesthesia type, use of a ureteral access sheath [UAS], insertion of a postoperative ureteral stent), and postoperative characteristics (length of hospital stay, postoperative complications, stone composition, and SFS during follow-up).

The preoperative stone burden was determined via computed tomography imaging in all patients. Stone location was classified as ureteric, pelvic ureteric junction, renal pelvis, or renal. For stones in multiple sites (ie, renal and ureteric stones, multiple calyces, or staghorn stones), the location was classified as renal. SFS was defined as the presence of only stone fragments <2 mm on completion of the procedure, confirmed endoscopically, with no evidence of stone fragments >2 mm on kidney-ureter-bladder X-ray or ultrasound imaging at 3-mo follow-up. Eight core preoperative parameters were identified as inputs for analysis: age, sex, preoperative urine culture result, anatomic variants, stone location, presence of multiple stones, total stone size, and preoperative urinary drainage (stent or nephrostomy). These inputs were used to predict four intraoperative or postoperative outcomes: UAS use, insertion of a postoperative stent, complications, and SFS.

### 2.2 Data preprocessing and analysis

Our workflow included data cleaning and preprocessing steps. The data were first cleaned, which included removal of blank spaces and irrelevant characters. Subsequent preprocessing involved imputation of mode values for preoperative characteristics because of their categorical nature. Then statistical analysis was conducted, encompassing correlation, variance inflation factor (ViF) assessment, and logistic regression analysis for all four tasks. In the next step, individual ML algorithms were trained for each task, and a multitask artificial neural network (ANN) was concurrently trained on all tasks collectively. Finally, explainable AI techniques were applied to provide explanations for the predictions generated by the algorithms.

### 2.3 ML algorithms

Sixteen ML algorithms were selected and trained using the data processed for each of the four tasks. The algorithms were logistic regression, quadratic discriminant analysis, extra trees classifier, Adaboost, gradient boost, extreme gradient boost (XGBoost), cat boost classifier, naïve Bayes, bagging classifier, support vector machine (SVM; linear, polynomial, and radial basis function kernels), decision tree, K-nearest neighbour (KNN), random forest, and linear discriminant analysis. Training for prediction of all eight input characteristics was performed for each classification algorithm for the four tasks individually: UAS use, insertion of a postoperative stent, complications, and SFS.

### 2.4 Multitask network and evaluation metrics

ML multitasking involve algorithms capable of making multiple predictions simultaneously. In this study, a multitask ANN was developed to predict all postoperative characteristics simultaneously. The network features shared layers for initial feature extraction, followed by task-dependent layers. The ANN includes an input layer with eight neurons, succeeded by a common hidden dense layer containing 128 neurons with a rectified linear unit (ReLU) activation function. Subsequently, the network diverges into four dense output layers, each consisting of one neuron with a sigmoid activation function, dedicated to predicting a specific post-operative characteristic.

The primary metrics used to assess the performance of ML algorithms are a confusion matrix and a classification report. A confusion matrix includes values for true-positive, true-negative, false-positive, and false-negative results. Metrics derived from a confusion matrix include accuracy, precision, recall, and the F1 score. A classification report provides precision and recall values for each class within the task.

### 2.5 Explainability

The significance of interpretability and explainability in networks has grown considerably, driven by heightened awareness and demand, particularly in critical application areas such as health care. Interpretability and explainability are needed when dealing with complex and less transparent predictive algorithms such as ANNs, which are often regarded as black boxes. Various techniques can be used to enhance understanding of the predictions generated by such models by emphasising the key features and their contributions to the output. We used tree explainer, feature importance, and Shapley additive explanation (SHAP) plots to illustrate explanations for the model predictions.

## 3 Results

### 3.1 Cohort demographic and operative results

A total of 872 patients underwent fURSL for urolithiasis during the study period. Males accounted for two-thirds of the cohort and the mean age was 57.37 (range 18–96) yr. Almost 90% of the cases had an acute presentation for renal colic, urosepsis, or haematuria, with the remainder diagnosed with kidney stones incidentally or during follow-up protocols. Preoperative drainage with insertion of a ureteric stent was needed in 32.9% of patients. At imaging, 35.55% of the subjects had a single stone, while 64.45% had two or more calculi. The mean stone burden was 12.70 ± 6.7 mm. A preoperative urine culture was routinely performed, and a positive urine culture requiring targeted therapy was observed in 12.73% of the patients.

Intraoperatively, an access sheath of variable size was used in 36.24% of the cases, while a postoperative stent was deemed necessary for 80.39% of the procedures. More than 90% of the patients had an uneventful postoperative course. All procedures were performed on a day-case basis, except for a short hospital stay for cases with comorbidities requiring observation or those with social issues. Of the patients who developed postoperative complications, 23 (2.4%) were readmitted for a urinary tract infection or urosepsis requiring antibiotics, and 21 for pain management.

At 3-mo follow-up, 92.66% of the patients had achieved SFS as confirmed by postoperative imaging of choice. Table 1 summarises the demographic, clinical, and procedural characteristics.

**Table 1:** Demographic, clinical, and procedural characteristics

Parameter	Result
Mean age, yr (standard deviation)	57.37 (19.47)
Sex, *n* (%)	
Male	553 (63.42)
Female	319 (36.58)

Presentation, *n* (%)	
Acute	772 (88.53)
Incidental/at follow-up	100 (11.47)

Number of calculi, *n* (%)	
1	562 (64.45)
≥2	310 (35.55)
Mean stone burden, mm (standard deviation)	12.70 (6.7)

Preoperative drainage, *n* (%)	
No	585 (67.09)
Yes	287 (32.91)
Positive preoperative urine culture, *n* (%)	111 (12.73)
Access sheath, *n* (%)	316 (36.24)
Postoperative JJ stent, *n* (%)	701 (80.39)

Complications, *n* (%)	
None	828 (94.95)
Pain	21 (2.41)
Urinary tract infection/sepsis	23 (2.64)
Stone-free status, *n* (%)	808 (92.66)

**Fig. 1:**
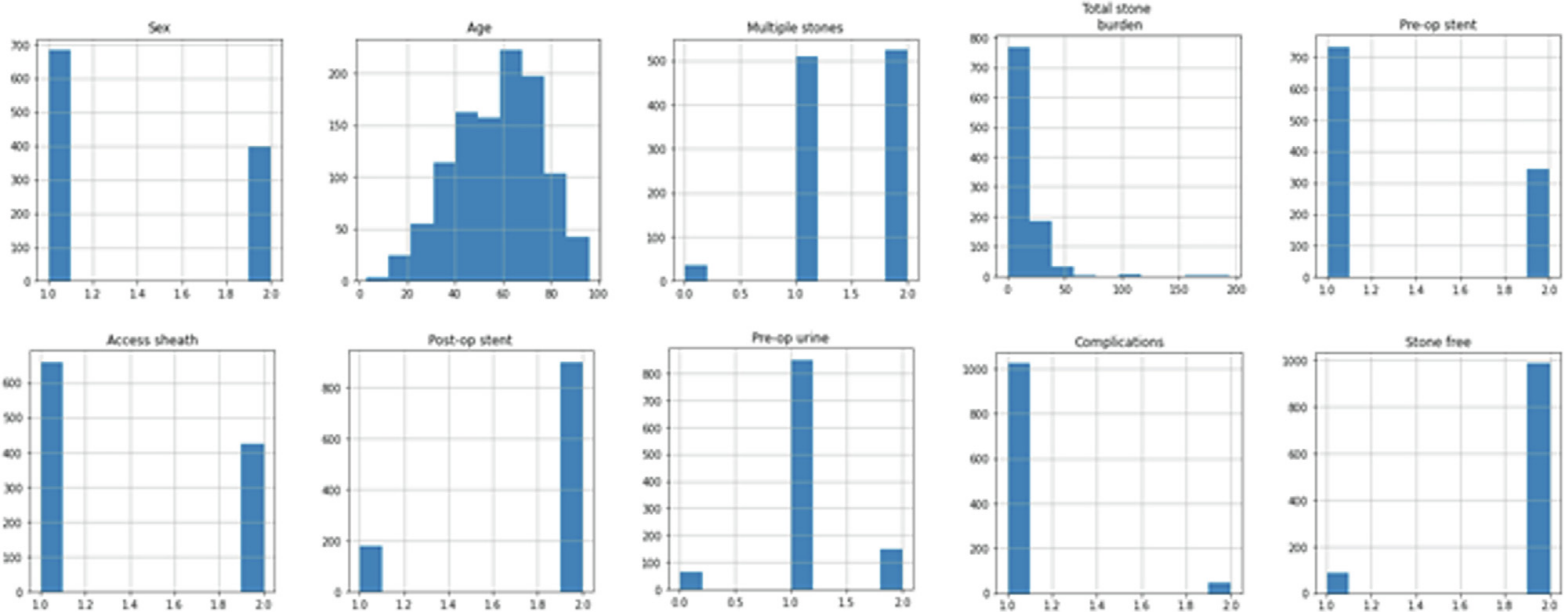
Histograms of the data distribution for all input features and the output predictors. Pre-op = preoperative; Post-op = postoperative.

Data analysis revealed that most of the variables were categorical in nature. Age was the only continuous variable, for which a symmetric distribution without any skewness was observed (Fig. 1). There were several outliers for total stone burden and some outliers for preoperative urine, total stone burden, complications, and SFS. A correlation heatmap and a box plot for all the input features and target predictors are provided in Supplementary Figures 1 and 2, respectively.

### 3.2 Prediction of UAS use

On correlation analysis, stone location showed the highest correlation (0.331), followed by total stone burden (0.2416) and multiple stones (0.1944). The effect of SFS was irrelevant (−0.0866). The other features showed much less correlation with the target variable.

ViF was evaluated to assess multicollinearity among the input features via a general analysis for all four dependent task vectors. All the output vectors had a high ViF, indicating good correlation between the other feature vectors. For example, use of a postoperative stent could strongly influence both SFS and the risk of complications.

Logistic regression was used to quantify the relationship between the input features and the output predictors. Patient sex (0.5) and a positive preoperative urine culture (0.42) had a strong positive impact on UAS use. The side on which the stone was located (−0.11) and the presence of a preoperative stent (−0.10) had a negative impact.

**Fig. 2:**
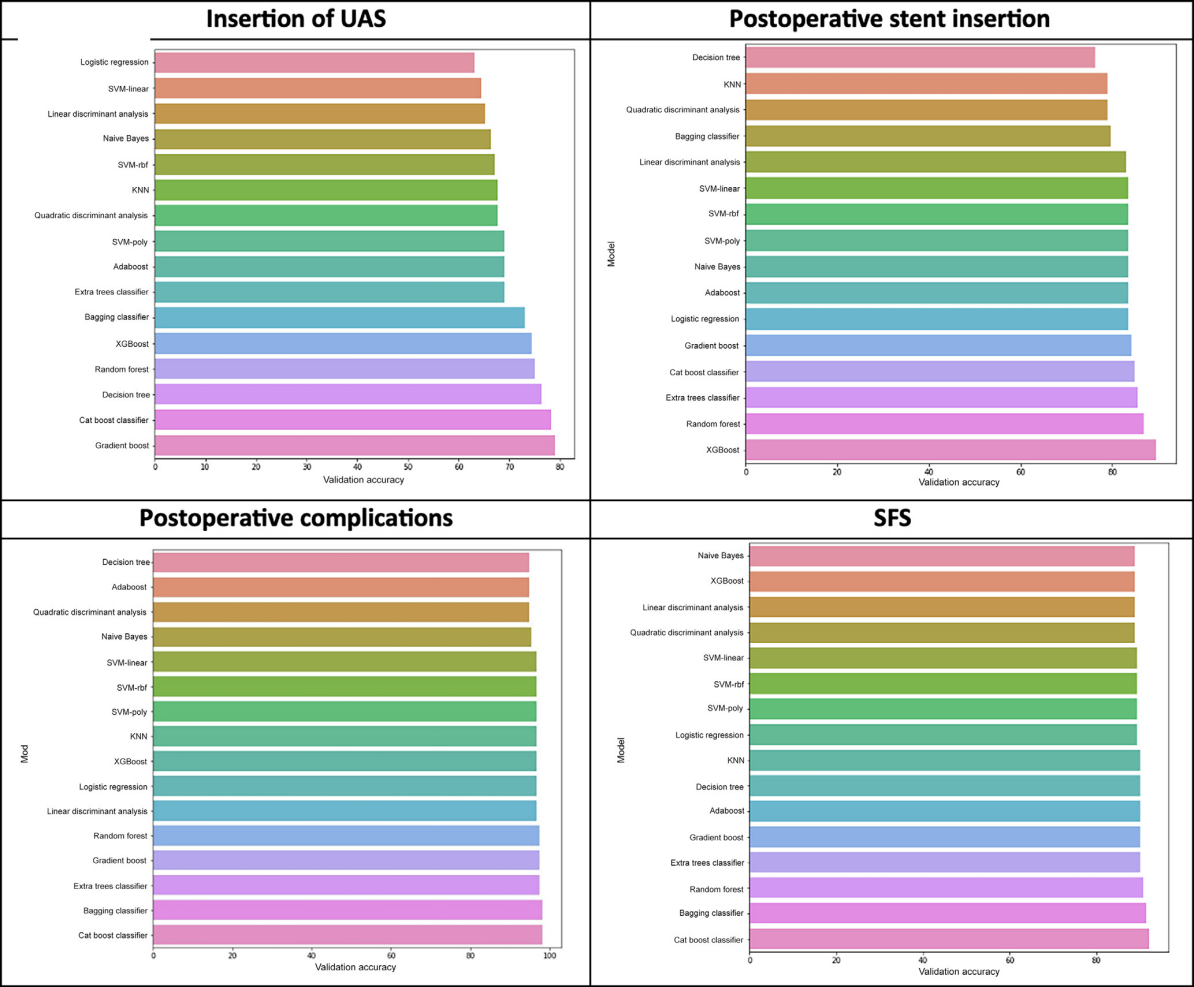
Bar plots comparing the validation accuracy of all the trained models for the four outcomes. SFS = stone-free status; UAS = ureteral access sheath. SVM = support vector machine; poly = polynomial; rbf = radial basis function; KNN = K-nearest neighbour; XGBoost = extreme gradient boost.

The 16 different ML algorithms were trained and tested for prediction of UAS use (Fig. 2). Gradient boost had the highest validation accuracy, followed by the cat boost and decision tree classifiers. The extra trees classifier had the highest precision and recall. Additional information (confusion matrices, classification reports, and precision recall) for the three best-performing ML algorithms for this task in the validation set is provided in Supplementary Figure 3.

Feature importance and SHAP techniques were used for explainable results on prediction of intraoperative UAS use (Supplementary Fig. 4). Total stone burden, stone location, and patient age were positively correlated with UAS use. In particular, greater age and total stone size were associated with greater UAS use, as were stones located in multiple calyces and staghorn stones (classified with higher values). The opposite was observed for a positive preoperative urine culture and female sex.

### 3.3 Prediction of ureteric stent insertion

Correlation analysis was conducted to qualitatively assess the relationship between the input features and the need for a postoperative stent. Age had the highest positive correlation (0.186), followed by multiple stones (0.11); the other parameters exhibited almost invariant correlation.

Logistic regression revealed that parameters with a negative impact on postoperative stent placement were the presence of a preoperative stent (0.5) and a negative urine culture (0.16). Parameters with a positive impact were female sex (0.43) and the presence of multiple stones (0.42).

Among the ML algorithms, XGBoost yielded the highest results in the validation set, followed by the random forest and extra trees classifiers (Fig. 2). Additional information on the three best-performing ML algorithms for this task in the validation set is provided in Supplementary Figure 5.

Explainable AI SHAP plots were used to depict the results for prediction of postoperative stent insertion (Supplementary Fig. 6). Total stone burden, patient age, and the presence of multiple stones had the strongest influence on postoperative stenting, and higher values had greater importance. Conversely, preoperative drainage was negatively correlated with postoperative stent insertion.

### 3.4 Prediction of complications

A positive preoperative urine culture (0.182) showed the highest correlation with the occurrence of postoperative complications. Correlation was weak for the presence of a preoperative stent (0.033) and other parameters.

Logistic regression revealed a strong positive impact of a positive urine culture (1.44), followed by female sex (0.46). Negative impacts were observed for total stone burden (−0.022) and insertion of a postoperative stent (−0.014).

Analysis revealed that the cat boost classifier yielded the best results in the validation set, with less overfitting (Fig. 2). The bagging and gradient boost classifiers were the second and third best-performing algorithms. Additional information on the performance of these three algorithms for this task in the validation set is available in Supplementary Figure 7.

**Fig. 3:**
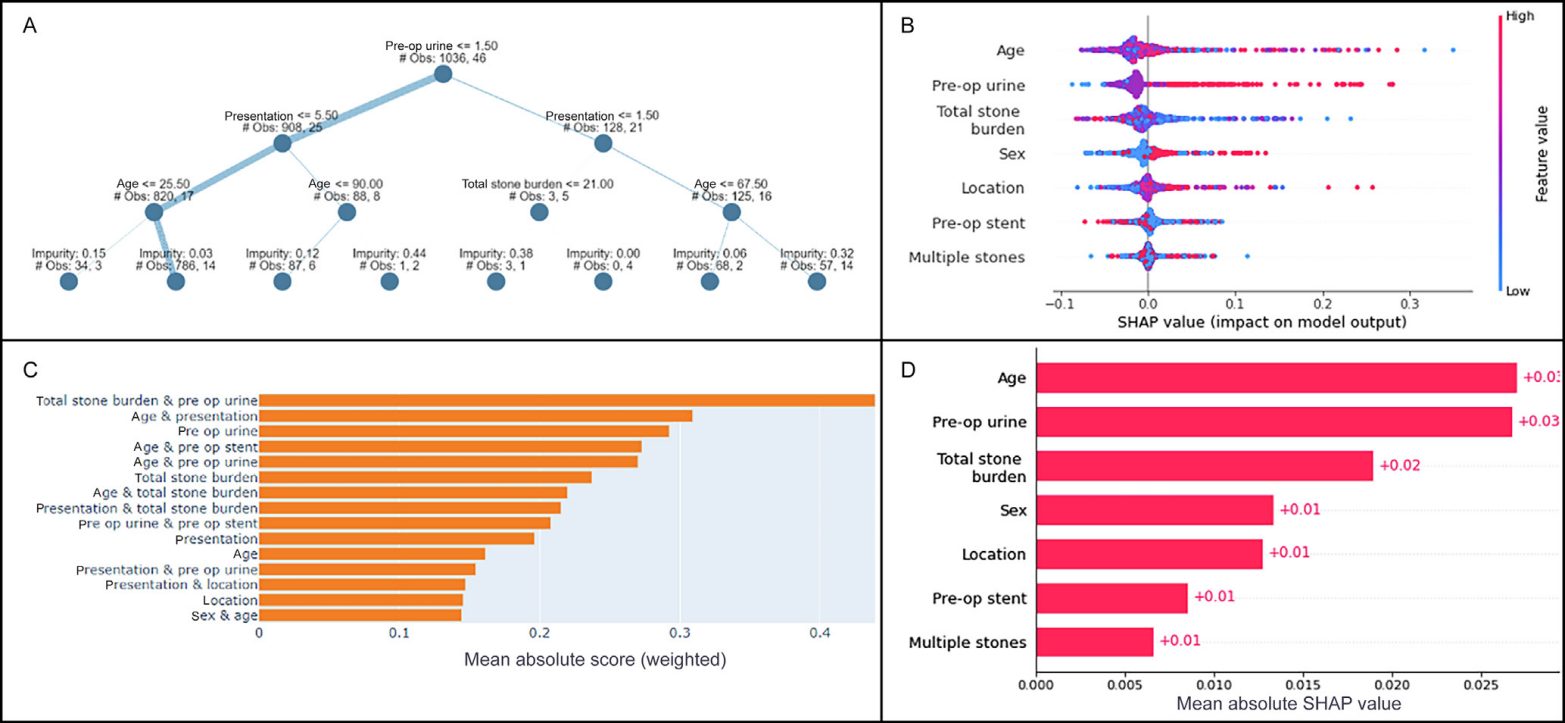
Explainable artificial intelligence results for the development of postoperative complications. (A) Explainable tree, (B) SHAP bee swarm plot, (C) bar chart of absolute SHAP scores, and (D) mean absolute SHAP values. SHAP = Shapley additive explanation; #Obs = number of observations; pre op = preoperative.

The correlation between postoperative complications and the other features is shown by the explainable AI results in Figure 3. Older age and a positive urine culture had a strong impact on the occurrence of complications, as well as a high total stone burden and acute presentation.

### 3.5 Prediction of SFS

Correlation with SFS was highest for total stone burden, with a value of −0.135; invariant correlation was observed for the other parameters.

Logistic regression revealed that failure to achieve SFS was positively impacted by total stone burden (0.34), followed by preoperative stent placement (0.106). Among the features negatively related to SFS failure, the highest result was for a negative preoperative urine culture (0.14), followed by stone location (0.09), including location in a single calyx or in the ureter.

Among the ML algorithms, the cat boost classifier had the best SFS prediction in the validation set, with accuracy of 93%, precision of 87%, and recall of 65%. The bagging classifier and random forest algorithm were ranked second and third for performance, so these three algorithms were used to elaborate the multitask predictive model. Additional information is available in Supplementary Figure 8.

**Fig. 4:**
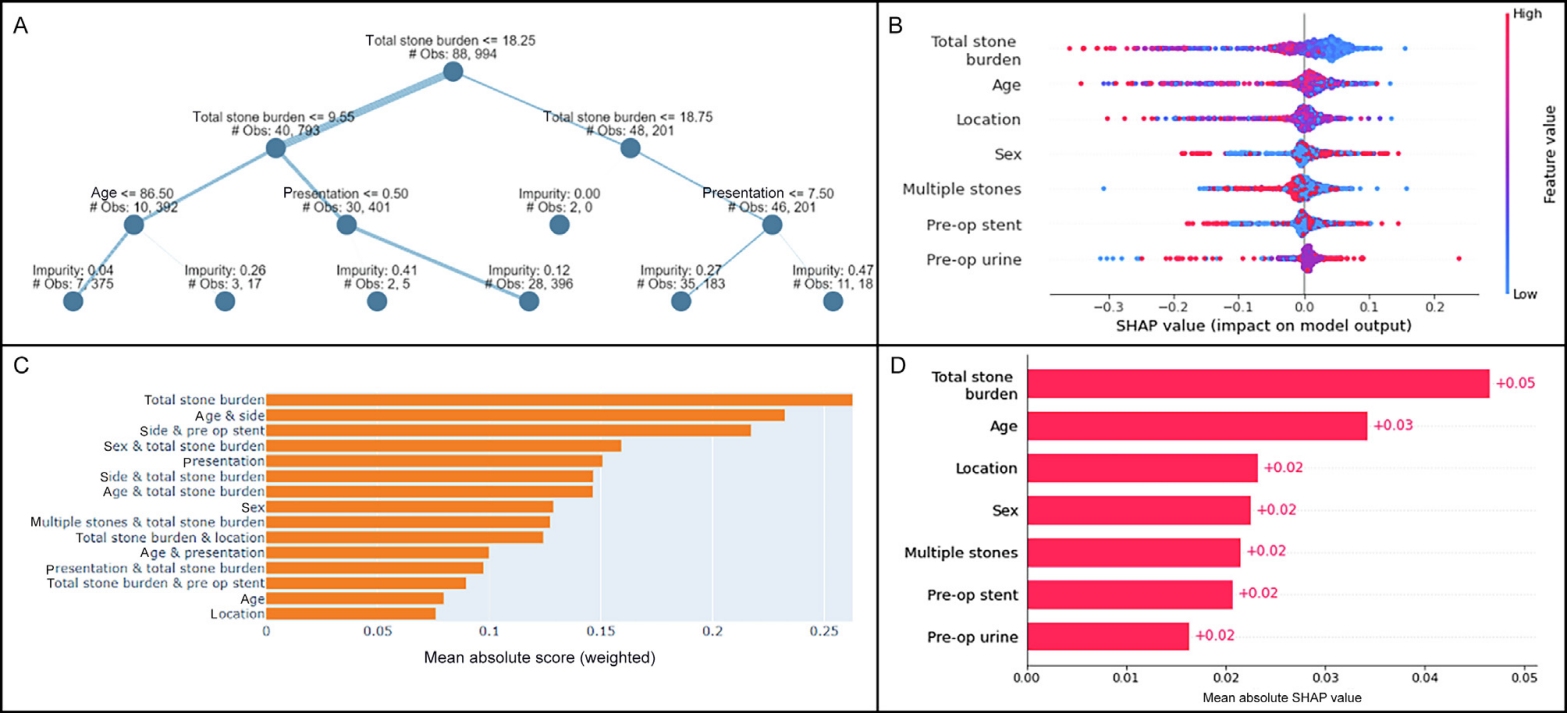
Explainable artificial intelligence results for stone-free status. (A) Explainable tree, (B) SHAP bee swarm plot, (C) bar chart of absolute SHAP scores, and (D) SHAP mean values. SHAP = Shapley additive explanation; #Obs = number of observations; pre op = preoperative.

Explainable AI results show the correlation between SFS and the other parameters. Total stone burden and patient age had the greatest influence on SFS, with older age and greater stone burden related to a lower likelihood of SFS (Fig. 4). Other parameters with a positive impact on failure to achieve SFS were elective presentation, male sex, and presence of a single stone.

## 4 Discussion

This study represents an initial exploration of ML use for predicting fURSL outcomes in adults with urolithiasis. We illustrate the capability of ML algorithms to accurately predict clinical outcomes using data for preoperative parameters, with a specific focus on stone burden and placement of a preoperative stent.

Our study findings demonstrate that SFS was correlated with stone burden and a positive preoperative urine culture, which is consistent with the literature [12]. If further studies confirm our results, ML could soon become part of the routine workflow to aid in surgical planning, balancing risks and benefits, and patient counselling. Our results show that a larger overall stone size was associated with lower likelihood of achieving SFS. This aligns with prior results emphasising the precision of overall stone size in determining outcomes [13]. However, consideration of the methodological variations for measurement of stone volume and the parameters that affect stone burden, such as number and shape, is essential. Recent technological advances, particularly those driven by AI, have revolutionised the medical field, with ML methods increasingly prevalent and adopted across urological subspecialties [14]. The role of radiomics and automated recognition of stone surface and dimension features is attracting increasing attention, with positive results for preoperative planning and prediction of possible outcomes [15].

We included the presence of a preoperative drain as an input feature and the decision to leave a postoperative stent as an output feature. Stent insertion before URS is the subject of debate [16] and our exploration of its impact in predicting surgical outcomes was merely observational in nature. In cases involving acute presentation with urosepsis and obstruction, stent insertion is non-negotiable and leads to postponement of fURSL. However, in borderline situations, the decision to insert a stent may impact subsequent outcomes [17]. Some authors noted that preoperative stenting can enhance the fURSL success rate by inducing “passive ureteric dilatation”, thereby reducing the risk of procedure failure [18]. The likelihood of requiring a postoperative stent is lower for patients with a preoperative drain. Conversely, studies investigating the correlation between indwelling stents and postoperative complications revealed that the risk of urinary tract infection after fURSL is significantly higher for patients with a preoperative indwelling stent for >2 mo [19], and the decision to stent instead of performing primary URS is not always cost-effective [20].

Previous studies on ML in kidney stone disease echo our findings. ML frameworks for prediction of outcomes such as hydronephrosis and septic events after fURSL or PCNL have shown impressive accuracy. Blum et al [21] used renogram data to develop an ML framework to improve early identification of clinically significant hydronephrosis caused by pelvic ureteric junction obstruction. They achieved noteworthy accuracy of 93% in predicting early detection of severe cases requiring surgical intervention. Pietropaolo and colleagues [22] used ML algorithms to predict septic events after fURSL in 114 patients and achieved accuracy of 81.3%. Aminsharifi et al [23] analysed data for 146 adult patients undergoing PCNL to evaluate the effectiveness of an ML algorithm in predicting outcomes and achieved impressive accuracy of up to 95%.

Our investigation reveals the proficiency of ML algorithms in managing intricate and multifaceted data sets, which is an advantage for prediction of postoperative results [24]. By using patient data, including demographic and preoperative characteristics, ML models can generate personalised risk assessments. These predictive models offer clinicians valuable insights into potential complications so that proactive measures can be taken to mitigate risks and improve patient recovery [25]. Integration of ML algorithms in health care systems can equip clinicians with decision support tools for preoperative planning and risk management. Predictive models can aid in identifying high-risk patients, tailoring postoperative care plans, and optimising resource allocation [26]. This can help health care providers to make informed decisions that enhance overall patient safety and optimise resources.

For the primary aim of optimising health care strategies, ML has been applied in other specialities for prediction of outcomes [27]. ML models can be trained and formalised by feeding the underlying algorithms with large amounts of real-life data. Such data distribution can be used to optimise the ML learning and training process, resulting in highly accurate regression coefficients and prediction outputs [25]. The efficiency of this approach has been demonstrated for modelling the survival time for melanoma patients on the basis of stage and demographic data, the risk of postpartum haemorrhage on admission to the labour ward [28], and the probability of successful treatment for various pathologies such as infectious diseases [29], diabetes [30], hypertension, and depression [31]. The number of possible applications of ML predictive models in health care is countless, and increasing interest and exploration in this field are likely in the near future.

While our study has limitations, including the retrospective nature, the lack of data for operative time, and a need for confirmation in prospective trials, it contributes to the field by introducing an automated predictive model for fURSL outcomes. Despite the promise of ML in health care, challenges such as data privacy concerns, model interpretability, and algorithmic biases require careful consideration to ensure responsible use in postoperative care.

## 5 Conclusions

URSL is a safe and efficient procedure for treatment of urolithiasis. With recent advances overcoming the classic limits of the procedure, there is a need to correctly predict surgical outcomes and the risk of developing complications. By training ML algorithms for these tasks, we were able to identify total stone burden and stone number, size, and location as the main risk factors for incomplete stone clearance. While the role of preoperative and postoperative stenting remains controversial, the likelihood of untreated urinary tract infections strongly correlates with postoperative complications. Our ML model achieved accuracy of >90% in predicting SFS and the incidence of complications, and lays a foundation for the development of an accessible predictive model for endourologists and patients in the fURSL setting.

  ***Author contributions***: Patrick Juliebø-Jones had full access to all the data in the study and takes responsibility for the integrity of the data and the accuracy of the data analysis.

  *Study concept and design*: Nedbal, Adithya, Naik, Gite, Juliebø-Jones, Somani.

*Acquisition of data*: Nedbal, Juliebø-Jones, Adithya, Naik, Gite.

*Analysis and interpretation of data*: Nedbal, Adithya, Naik, Gite.

*Drafting of the manuscript*: Nedbal, Somani.

*Critical revision of the manuscript for important intellectual content*: Nedbal, Adithya, Naik, Gite, Juliebø-Jones, Somani.

*Statistical analysis*: Adithya, Naik, Gite.

*Obtaining funding*: None.

*Administrative, technical, or material support*: None.

*Supervision*: Somani, Juliebø-Jones.

*Other*: None.

  ***Financial disclosures:*** Patrick Juliebø-Jones certifies that all conflicts of interest, including specific financial interests and relationships and affiliations relevant to the subject matter or materials discussed in the manuscript (eg, employment/affiliation, grants or funding, consultancies, honoraria, stock ownership or options, expert testimony, royalties, or patents filed, received, or pending), are the following: None.

  ***Funding/Support and role of the sponsor*:** None.
