# Supplementary material for: Can Machine Learning Correctly Predict Outcomes of Flexible Ureteroscopy with Laser Lithotripsy for Kidney Stone Disease? Results from a Large Endourology University Centre
Source: Eur Urol Open Sci. 2024 May 22;64:30–7. doi: 10.1016/j.euros.2024.05.004 (PMC11145425; doi:10.1016/j.euros.2024.05.004)
Supplement: Supplementary Data 1 [file mmc1.docx]

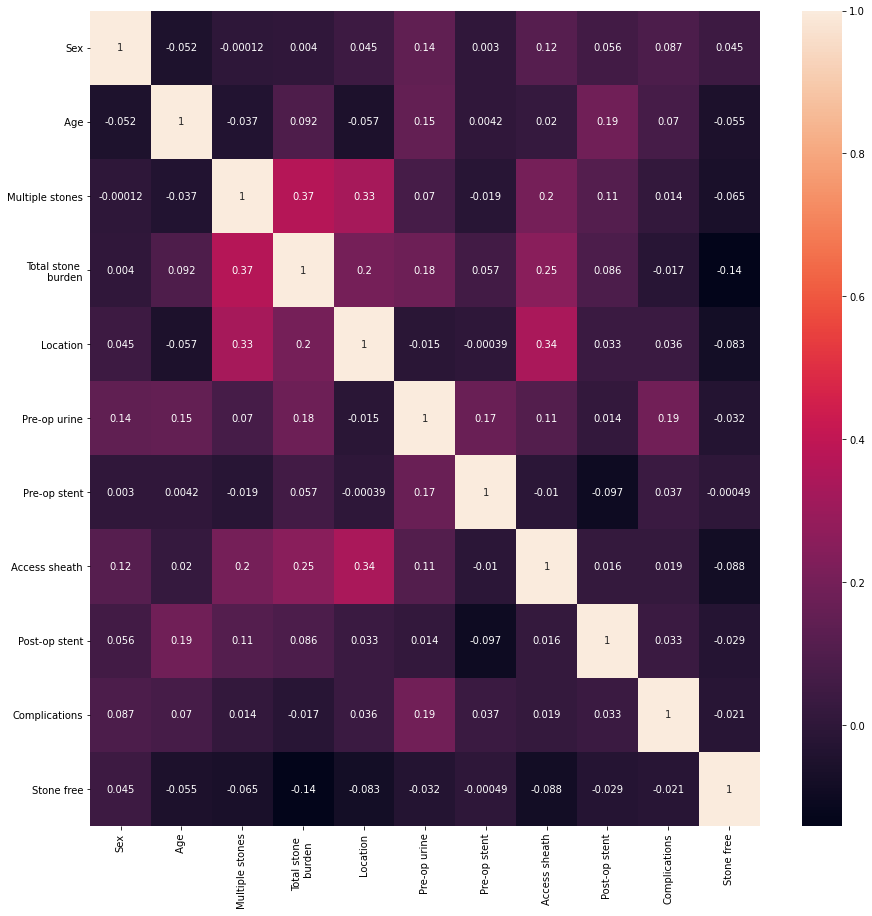


Supplementary Fig. 1 Correlation heatmap of all the input features and output predictors.


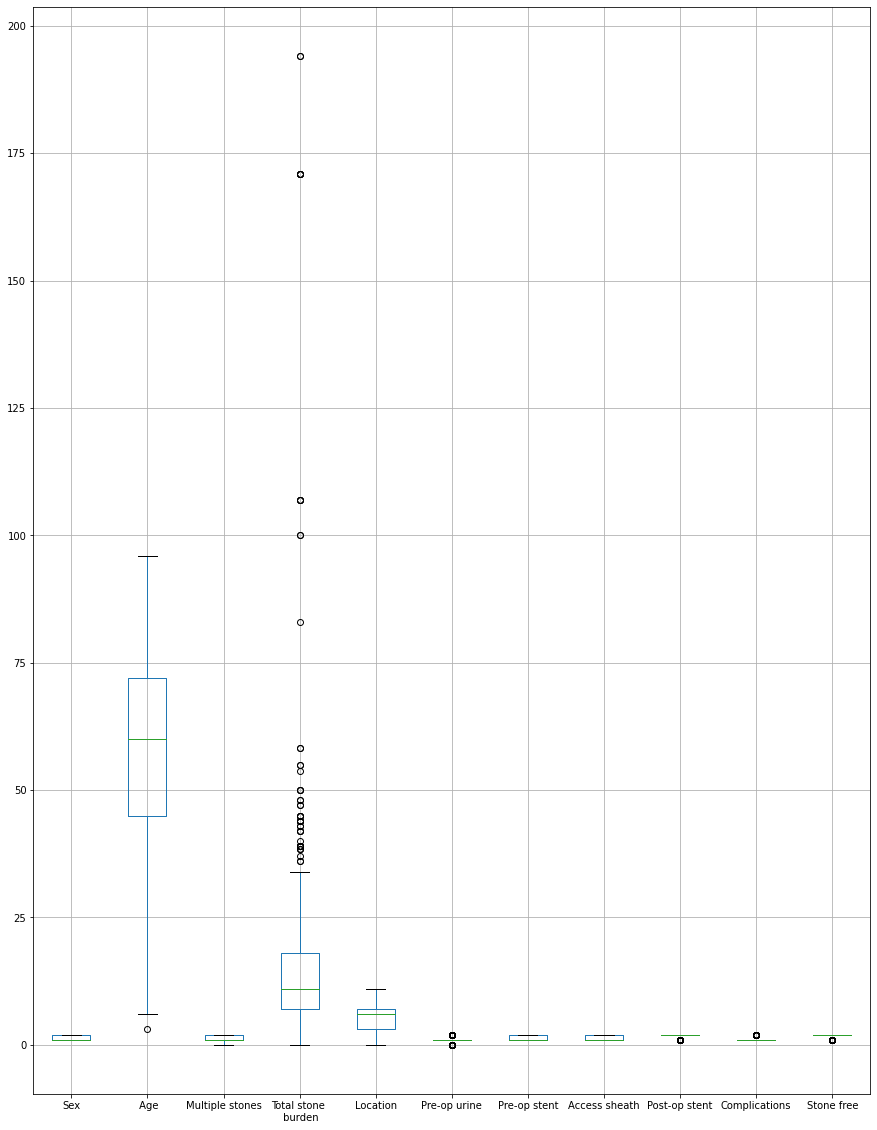


Supplementary Fig. 2 Boxplot for all the input features and target predictors.

| **Algorithm** | **Training** | **Validation** |
| --- | --- | --- |
| **Gradient boost** | **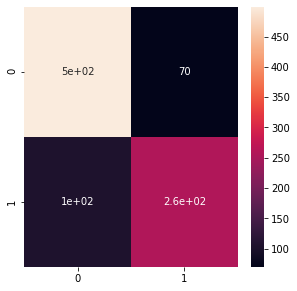** | **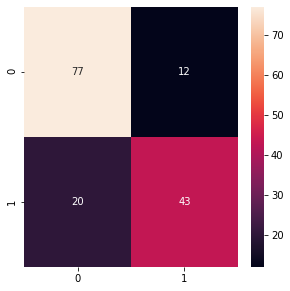** |
|  | **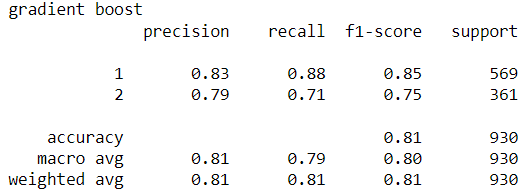** | **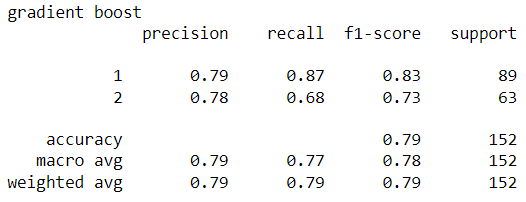** |
|  | **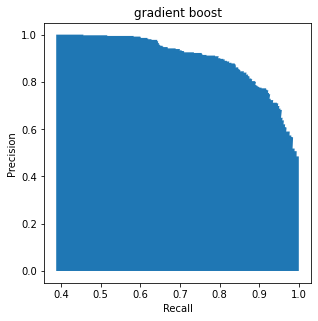** | **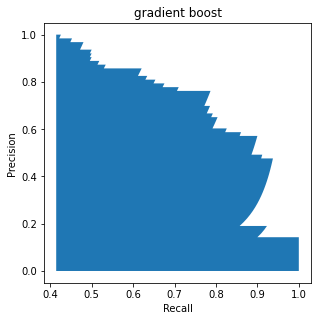** |
| **Cat boost classifier** | **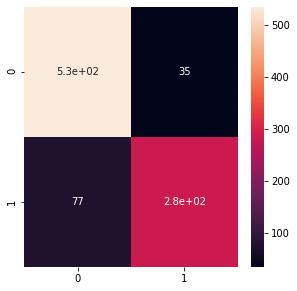** | **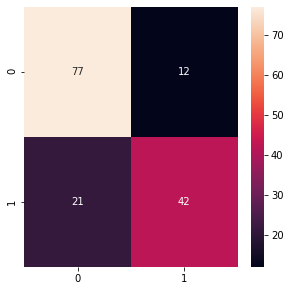** |
|  | **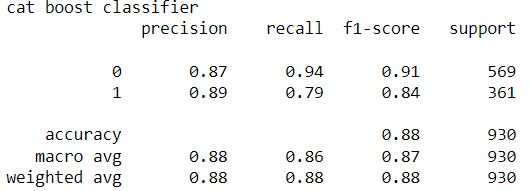** | **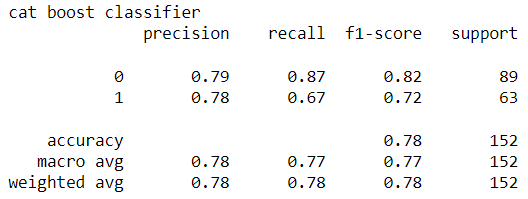** |
|  | **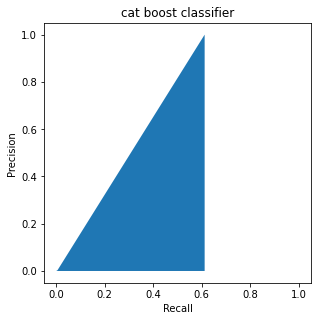** | **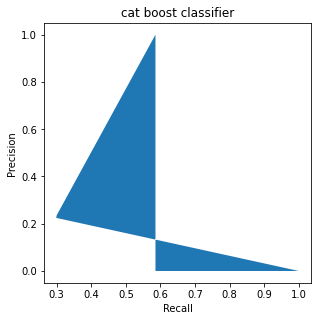** |
| **Decision tree** | **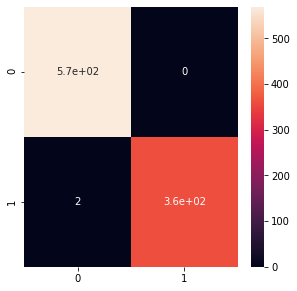** | **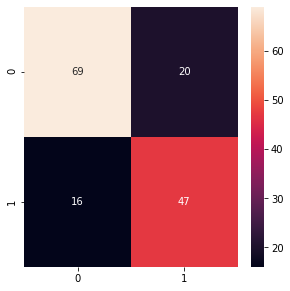** |
|  | **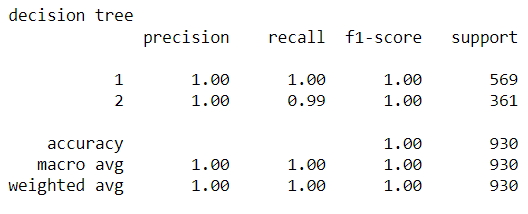** | **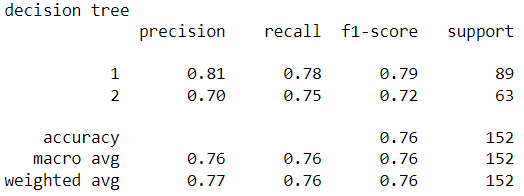** |
|  | **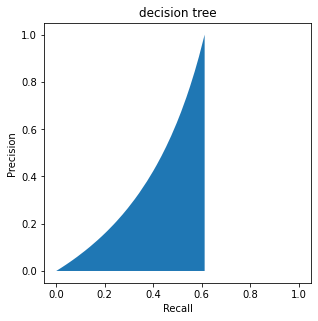** | **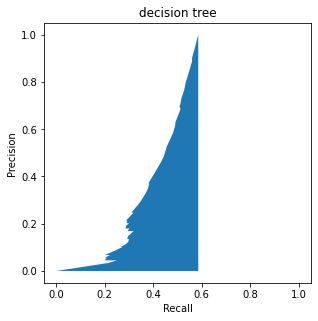** |

Supplementary Fig. 3 Confusion matrices, classification reports and precision recall curves of the machine learning algorithms for access sheath prediction.

| **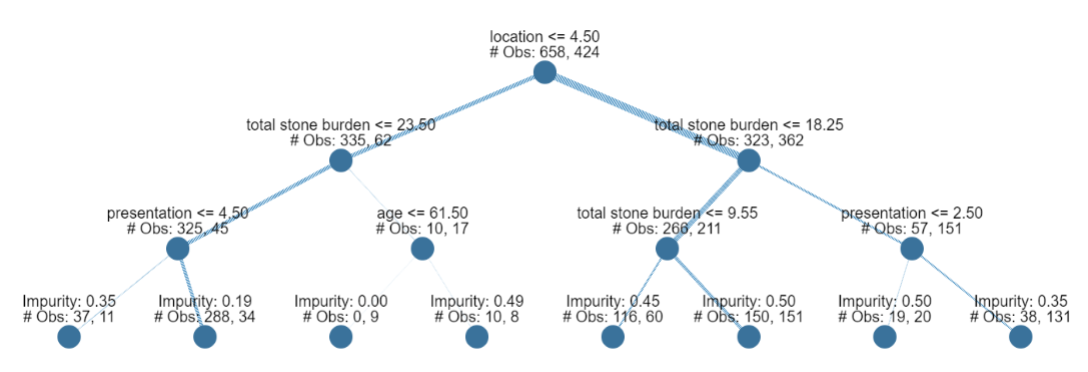** |
| --- |
| **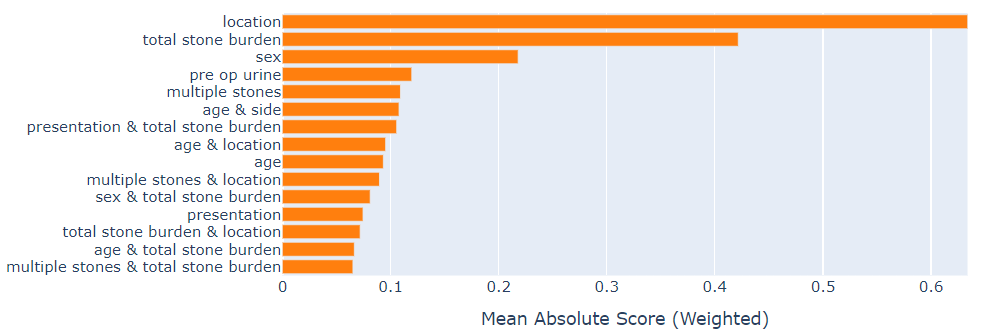** |
| 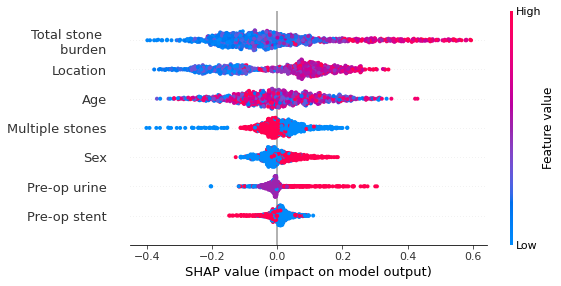 |
| 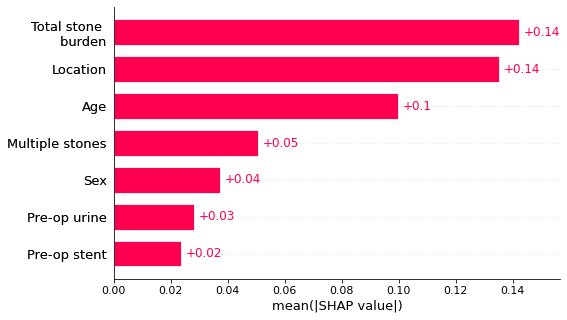 |

Supplementary Fig. 4 Explainable AI results for use of access sheath. From the top: explainable tree, shap bar chart for absolute scores, shao beeswarm, shap chart corrected for mean values.

| **Algorithm** | **Training** | **Validation** |
| --- | --- | --- |
| **XGBoost** | 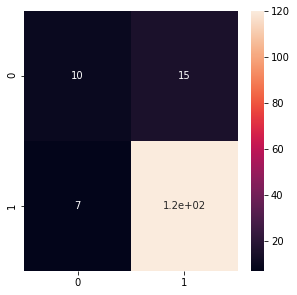 | **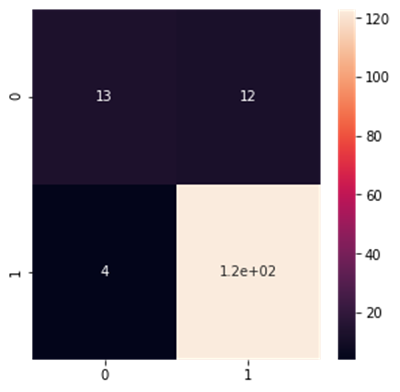** |
|  | 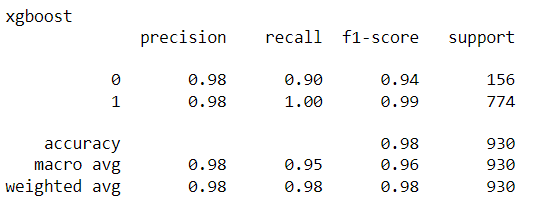 | 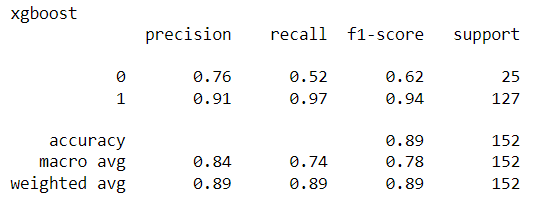 |
|  | **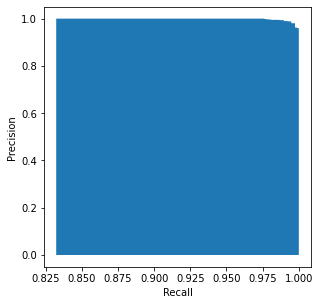** | **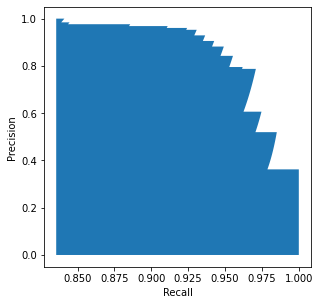** |
| **Random forest** | 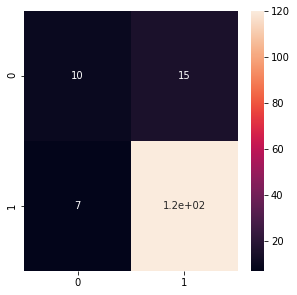 | 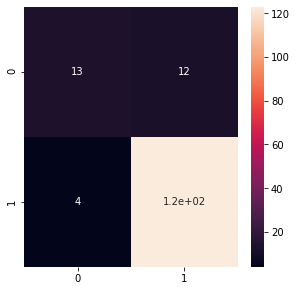 |
|  | 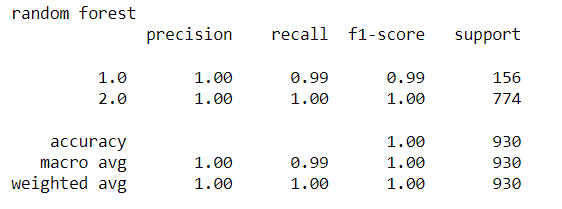 | 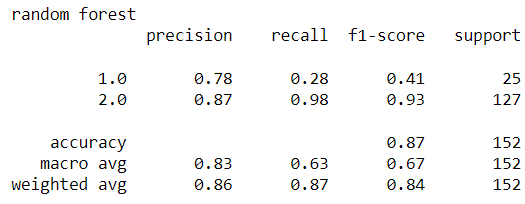 |
|  | **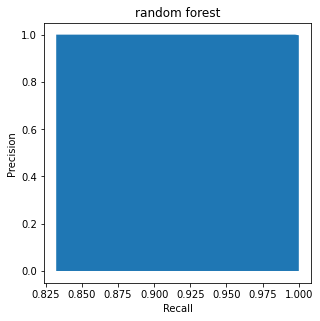** | **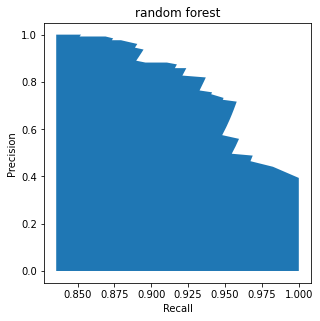** |
| **Extra trees classifier** | 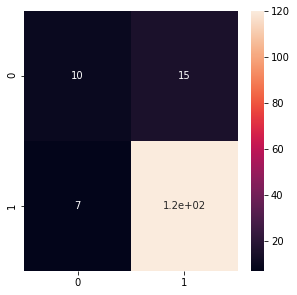 | 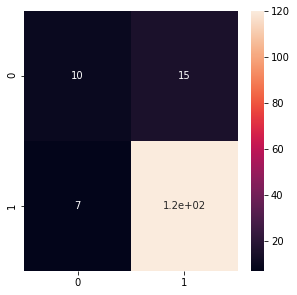 |
|  | 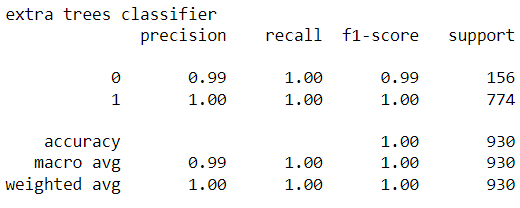 | 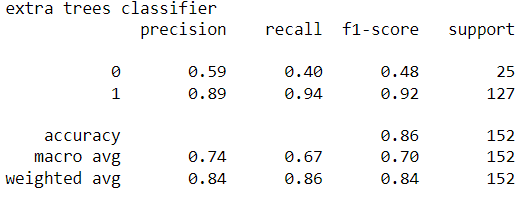 |
|  | **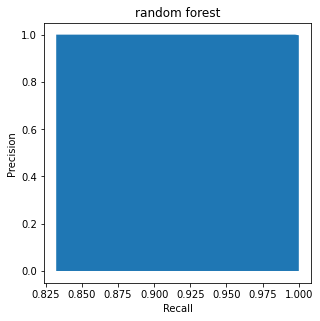** | **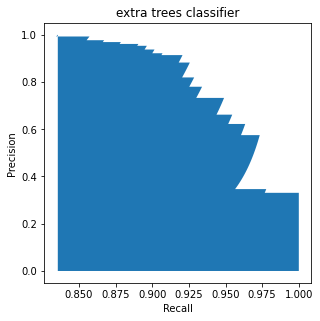** |

Supplementary Fig. 5 Confusion matrices, classification reports and precision recall curve of the machine learning algorithms for prediction of insertion of postoperative stent.

| **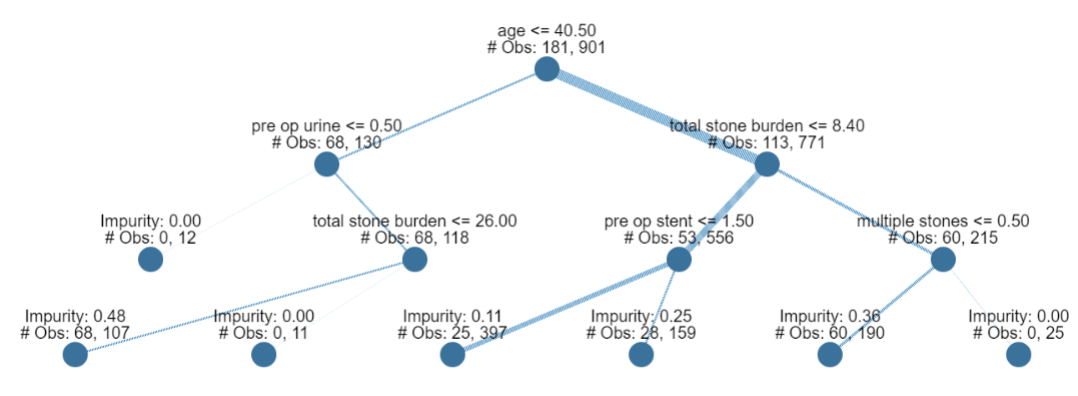** |
| --- |
| **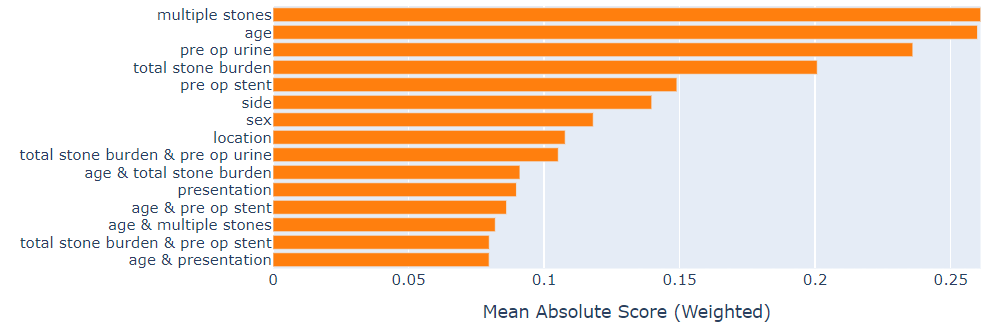** |
| 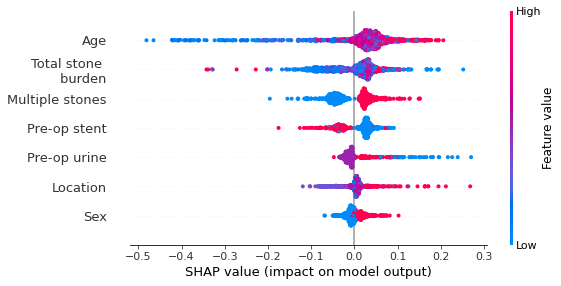 |
| 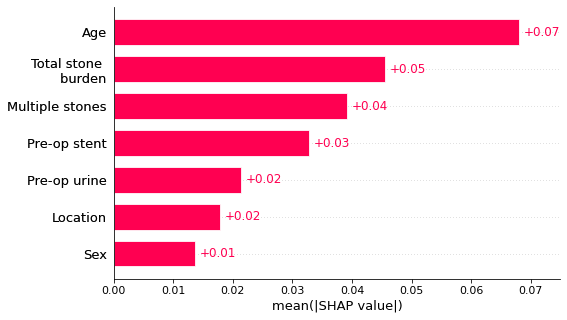 |

Supplementary Fig. 6 Explainable AI results for insertion of postoperative stent. From the top: explainable tree, shap bar chart for absolute scores, shao beeswarm, shap chart corrected for mean values.

| **Algorithm** | **Training** | **Validation** |
| --- | --- | --- |
| **Catboost** | 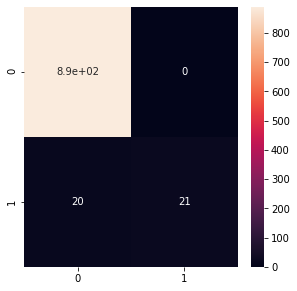 | 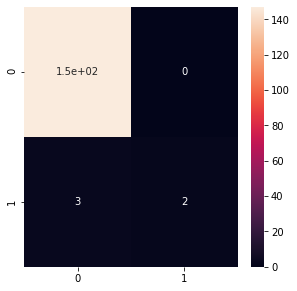 |
|  | 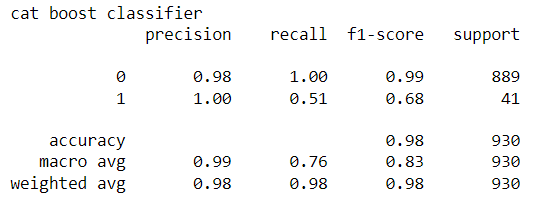 | 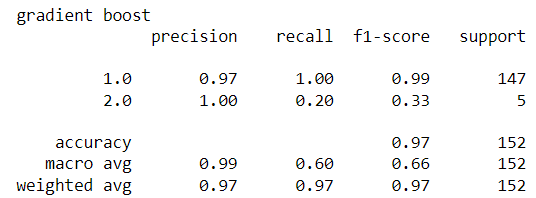 |
|  | 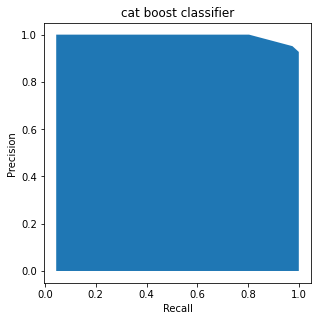 | 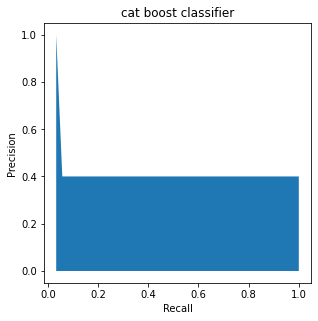 |
| **Bagging classifier** | 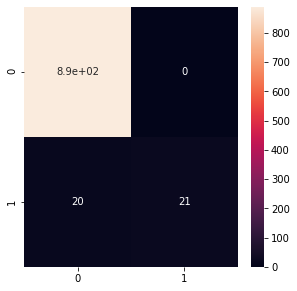 | 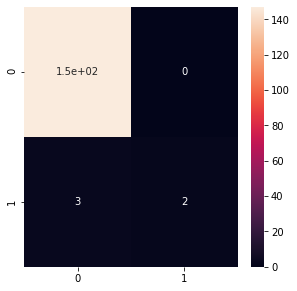 |
|  | 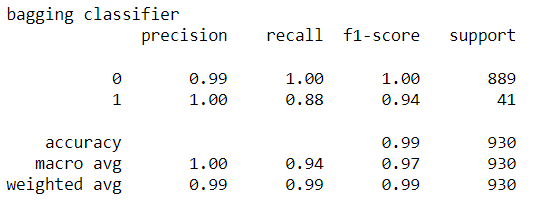 | 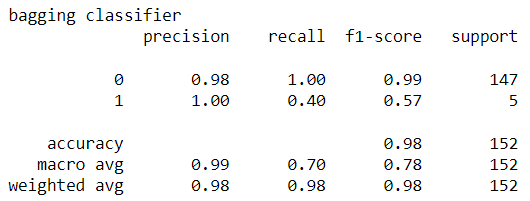 |
|  | 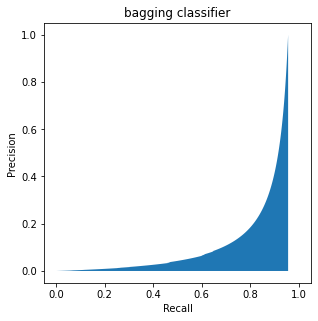 | 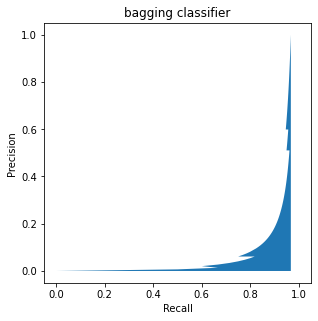 |
| **Gradient boost classifier** | 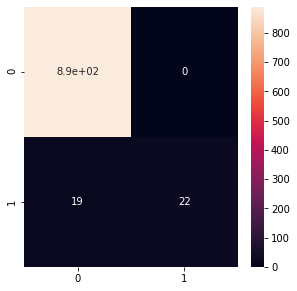 | 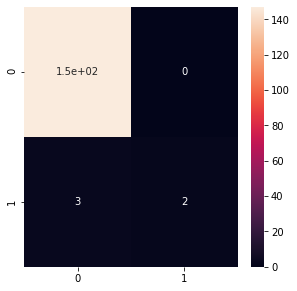 |
|  | 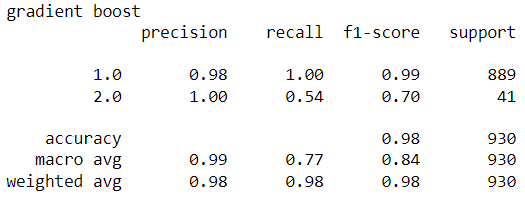 | 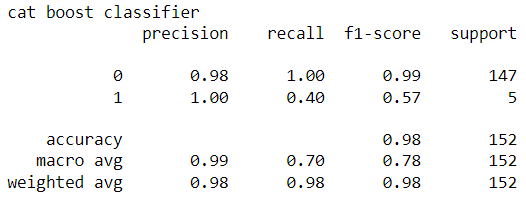 |
|  | 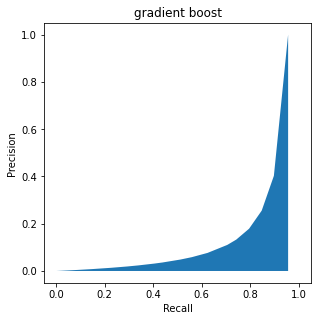 | 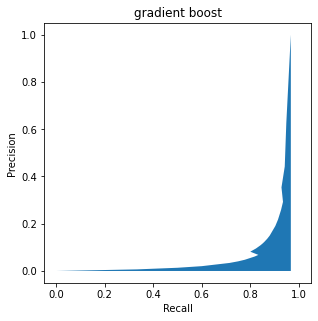 |

Supplementary Fig. 7 Confusion matrices, classification reports and precision recall curve of the machine learning algorithms for prediction of postoperative complications.

| **Algorithm** | **Training** | **Validation** |
| --- | --- | --- |
| **Catboost** | 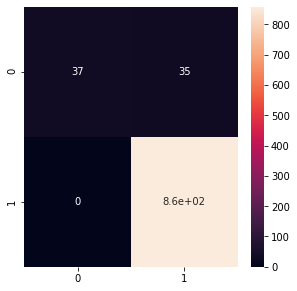 | 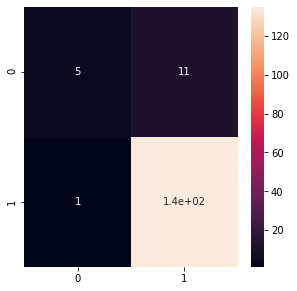 |
|  | 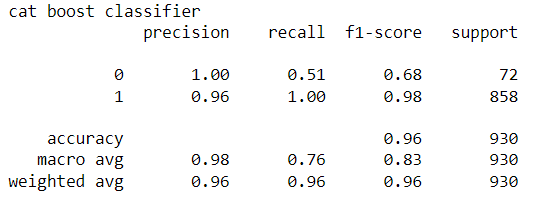 | 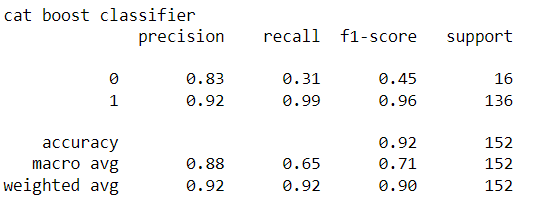 |
|  | 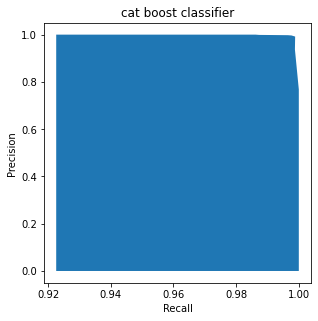 | 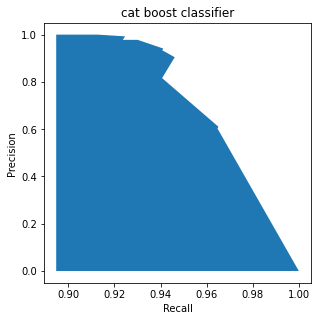 |
| **Bagging classifier** | 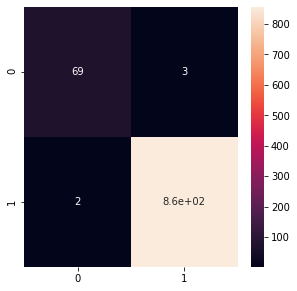 | 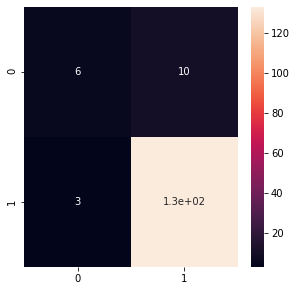 |
|  | 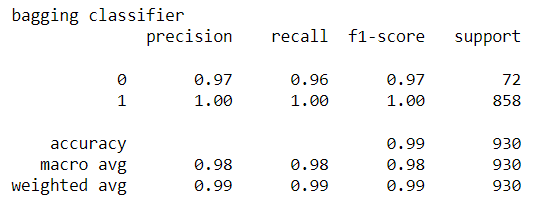 | 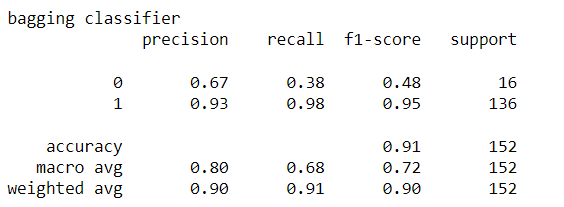 |
|  | 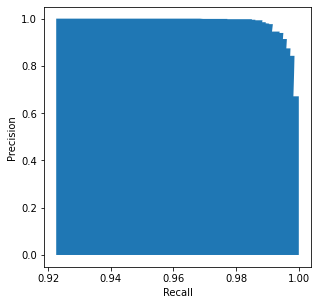 | 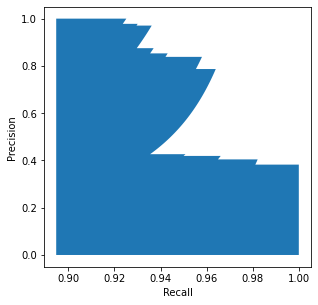 |
| **Random forest** | 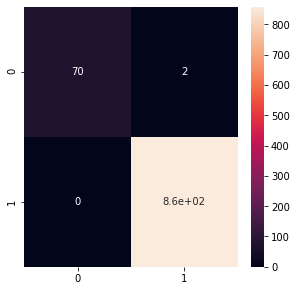 | 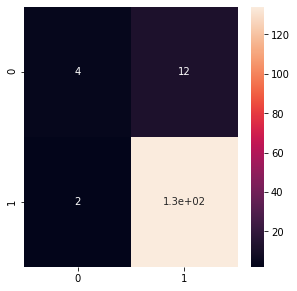 |
|  | 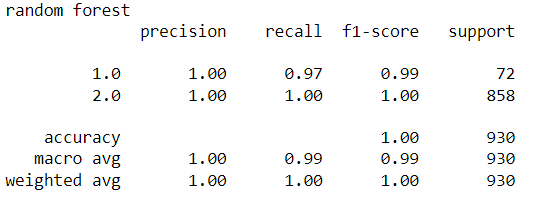 | 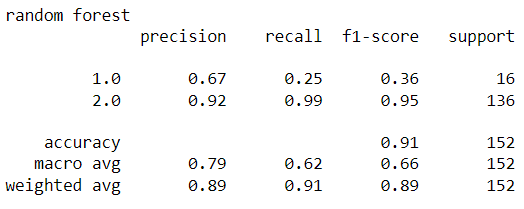 |
|  | 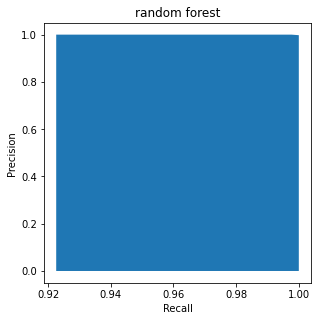 | 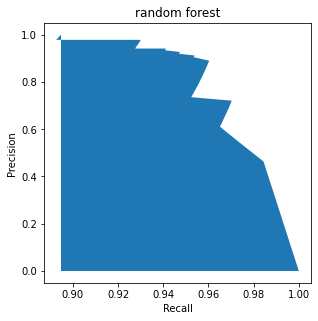 |

Supplementary Fig. 8 Confusion matrices, classification reports and precision recall curve of the machine learning algorithms for prediction of SFS.
